# The association between dietary antioxidant quality score with metabolic syndrome and its components in Iranian adults: A cross‐sectional study

**DOI:** 10.1002/fsn3.2067

**Published:** 2020-12-15

**Authors:** Mahshid Shahavandi, Hossein Shahinfar, Nastaran payande, Fatemeh Sheikhhossein, Kurosh Djafarian, Sakineh Shab‐Bidar

**Affiliations:** ^1^ Department of Community Nutrition School of Nutritional Sciences and Dietetics Tehran University of Medical Sciences (TUMS) Tehran Iran; ^2^ Students’ Scientific Research Center (SSRC) Tehran University of Medical Sciences (TUMS) Tehran Iran; ^3^ Department of Clinical Nutrition School of Nutritional Sciences and Dietetics Tehran University of Medical Sciences (TUMS) Tehran Iran

**Keywords:** antioxidants, blood pressure, metabolic syndrome, minerals

## Abstract

We aimed to investigate whether adherence to dietary antioxidant quality score (DAQS) is associated with metabolic syndrome (MetS) among the Tehranian population. This cross‐sectional study was conducted on 270 apparently healthy adults aged between 18 and 45 years old who lived in Tehran, Iran, between February 2017 and December 2018. Participants were categorized based on tertile cut‐off points of DAQs. To examine the association between DAQS and MetS and its components, we used multivariable logistic regression analysis in different models. Adherence to DAQS was associated with a significant increase for intake of vitamin B6 (P‐value = 0.02), riboflavin (P‐value < 0.001), folate (P‐value = 0.03), selenium (P‐value = 0.03), vitamin D (P‐value < 0.001), and calcium (P‐value < 0.001). Adherence to DAQS showed a significant decrease for odds of systolic blood pressure (SBP) (OR: 0.17, 95% CI: 0.04, 0.65, P‐value = 0.03). We also found that the overall adherence to DAQS was not significantly related to MetS and its other components. In conclusion, although we observed an improvement in SBP with a greater adherence to dietary antioxidant quality score, there was no association between DAQS and metabolic syndrome and its other components.

## Graphical abstract

We aimed to investigate whether adherence to dietary antioxidant quality score (DAQs) is associated with metabolic syndrome (MetS) among the Tehranian population. In conclusion, although we observed an improvement in SBP with a greater adherence to dietary antioxidant quality score, there was no association between DAQs and metabolic syndrome and its other components.

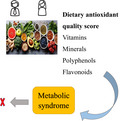

## 1 INTRODUCTION

The metabolic syndrome (MetS) refers to a series of metabolic disorders including lipid disorders, deviant homeostasis of glucose, abdominal obesity, and high blood pressure. All of these metabolic disorders are associated with an increased risk of diabetes mellitus, nonalcoholic fatty liver disease, chronic kidney disease, cardiovascular disease (CVD), stroke, some cancers, and even death (Ghorabi et al., 2020; Grundy, 2006). The etiology of MetS is not well understood, but a combination of genetic and environmental factors has been shown to lead to this syndrome (Poulsen et al., 2001; Shahinfar et al., 2020; Yosaee et al., 2017). Recent studies support the notion that these metabolic abnormalities do indeed cluster beyond the influence of chance and that the correlation may be subject to a single factor (Aizawa et al., 2007; Pladevall et al., 2006). There is increasing evidence that this systemic disorder can be a consequence of oxidative stress (Ceriello & Motz, 2004; Roberts & Sindhu, 2009). Nonetheless, oxidative stress plays an important role in vascular pathogenesis by either activating or exacerbating the biochemical processes that follow the metabolic syndrome (Shahavandi et al., 2020; Stocker & Keaney Jr, 2004). It was proposed that an impaired balance between free radical production and an impaired antioxidant defense system leading to oxidative damage may play a major role in pathological conditions such as insulin resistance, impaired energy production, and endothelial dysfunctions (Stocker & Keaney Jr, 2004). While dietary antioxidants have been confirmed to protect against oxidative damage and its complications, the effects of antioxidants on the risk of MetS and linked metabolic disorders have not been explained and the findings of clinical trials are inconsistent in this respect (Czernichow et al., 2006, 2009; Frei, 2004). The main source of exogenous antioxidants, such as vitamins, minerals, and polyphenols that help the body in removing excessive free radicals, are derived from diets (R. Sharma, 2014). An adequate antioxidant dietary intake promotes beneficial health outcomes. Individuals eat meals consisting of a variety of foods with complex antioxidant nutrient combinations but not isolated nutrients (Rivas et al., 2012). The most commonly used strategy for assessing the potential role of antioxidant dietary intake in health outcomes was based on individual nutrients (Rivas et al., 2012). This approach of considering the effects of a few single antioxidants on health outcomes lacks a great deal of information about the complex or cumulative relationships and interactions between the antioxidants in foods (Rivas et al., 2012). The Dietary Antioxidant Quality Score (DAQS), which summarizes certain dietary antioxidants and assigns a measured quantity score relative to the FDA recommended quantity, was developed to determine the overall effect of antioxidants on health outcomes (Rivas et al., 2012). We hypothesized that consumption of high dietary antioxidant quality following a healthy diet is associated with improvement in metabolic syndrome and its components. Therefore, we aimed to investigate the association between dietary antioxidant quality score and MetS among the Tehranian population.

## 2 SUBJECTS AND METHODS

### 2.1 Study design

This cross‐sectional study was conducted on 270 adults (118 males and 152 females), aged between 18 and 45 years’, old who lived in Tehran, Iran, between February 2017 and December 2018. Participants were recruited using advertisement, distribution of flyers in the common area, and information sessions held at residential facilities. The participants were selected based on the following inclusion criteria: 1) apparently healthy adult with age range of 18–45 years, 2) no alcohol or drug abuse, and 3) participants with special diets, such as weight loss or weight gain diets, adults with chronic diseases affecting the CRF, including diabetics, hormonal, and cardiovascular diseases, pregnant and lactating women, receiving any special medication or supplements (slimming medicine, hormone, sedative, supplements containing thermogenic substances, such as caffeine and green tea and linoleic acid conjugate) were excluded from the study. All procedures were followed in accordance with the ethical standards of the Tehran University of Medical Sciences (Ethic Number: IR.TUMS.VCR.REC.1396.4085), who approved all aspects of the study. All participants signed a written informed consent prior to the start of the study.

### 2.2 Anthropometric measures and body composition

Body weight was determined using a standard body weight scale (Seca 707; Seca GmbH & Co. KG., Hamburg, Germany). The patient's height was measured, unshod, using a stadiometer (Seca, Germany). To measure waist‐hip ratio (WHR), waist circumference in centimeters was divided by hip circumference in centimeters. We measured waist circumference (WC) between the middle of the bottom ribs and pelvic bones, after normal exhalation, using a nonstretch tape measure. Body mass index (BMI) was calculated as weight in kilograms, divided by height in meters squared. Body composition including fat‐free mass (FFM) and fat mass (FM) was measured using a body composition analyzer (InBody 720, Biospace, Seoul, Korea), where all patients were asked to follow these conditions before measurement: no food ingestion for at least 4 hr, minimal intake of 2 L of water the day before, no physical activity for at least 8 hr, no coffee or alcoholic beverage consumption during at least 12 hr, and no diuretic use for at least 24 hr, prior to assessment, respectively. Patients were required to urinate immediately before the body composition test (Korth et al., 2007).

### 2.3 Dietary assessment

The dietary intake of participants was assessed using a valid and reliable semiquantitative food frequency questionnaire (FFQ) (Mirmiran et al., 2010), which contained 168 food items. FFQ was administered by trained dieticians, via face‐to‐face interviews, asking participants to report their frequency of consumption of each food item, during the past year on a daily, weekly, or monthly basis. These reports were then converted to daily intakes. The food items were analyzed for their energy content using Nutritionist IV software, modified for Iranian foods (version 7.0; N‐Squared Computing, Salem, OR, USA).

### 2.4 Measurement of DAQS

Dietary antioxidant quality score (DAQS) was obtained from some vitamins and minerals that have antioxidant function including selenium, zinc, vitamin A, vitamin C, and vitamin E (Tur et al., 2005). To create a DAQS, we compared the daily intake of nutrients to that of the recommended daily intake (RDI) (Shils & Shike, 2006). Each of the five antioxidants intake was assessed and then we allocated a value of 0 or 1, separately, for every all components. When the intake was lower than 2/3 of the RDI, it was assigned a value of 0. Similarly, when the intake was higher than 2/3 of the RDI, it was assigned a value of 1. Thus, the total DAQS ranged from 0 (very poor quality) to 5 (high quality). According to Tur et al. method when the intake was lower than 2/3 of the RDI, it was assigned a value of 0. Similarly, when the intake was higher than 2/3 of the RDI, it was assigned a value of 1. Thus, the total DAQS ranged from 0 (very poor quality) to 5 (high quality) (Tur et al., 2005). The percentage of the RDI and the proportion of individuals with intakes below the RDI, 2/3 of the RDI, and 1/3 of the RDI were calculated. The proportion of individuals with intakes below 2/3 of the RDI was the criterion used to estimate the risk of inadequate intake (Aranceta et al., 2001).

### 2.5 Laboratory investigation

10 ml of blood and 3 ml of blood samples were obtained between the hours of 7 and 10 a.m. from all of fasted participants. Then blood samples were collected in acid‐washed test tubes without anticoagulant. After storing at room temperature for 30 min and clot formation, blood samples were centrifuged at 1,500 g for 20 min. Serums were stored in ‐ 80°C until future testing. Glucose was assayed by the enzymatic (glucose oxidase) colorimetric method using a commercial kit (Pars Azmun, Tehran, Iran). Serum total cholesterol (TC) and high‐density lipoprotein cholesterol (HDL‐C) were measured using a cholesterol oxidase phenol amino antipyrine method, and triglyceride (TG) was measured using a glycerol‐3 phosphate oxidase phenol amino antipyrine enzymatic method.

### 2.6 Definition of terms

MetS was defined according to the NCEP‐ ATP III classification as three or more of WC > 102 cm in males and WC > 88 cm in females, fasting plasma glucose ≥ 100 mg/dl in both gender, or a known diagnosis diabetes, fasting serum TG ≥ 150 mg/dl in both gender, fasting HDL cholesterol < 40 mg/dl in males and HDL < 50 in females, or blood pressure ≥ 130/85 mmHg in both gender (Aranceta et al., 2001).

### 2.7 Other measurements

Information on lifestyle was collected via self‐administered questionnaires, and included age (continues variable), sex (male or female), cardiovascular disease (yes or no), diabetes (yes or no), and smoking status (current, former, or never smoking), marital status (single or married) and physical activity was assessed using a validated short form of the International Physical Activity Questionnaire (IPAQ) (Moghaddam et al., 2012). Subjects were grouped into three categories including very low (<600 MET‐minute/week), low (600–3000 MET‐minute/week), moderate and high (>3,000 MET‐minute/week), which were calculated based on metabolic equivalents (METs) (Wareham et al., 2003).

### 2.8 Statistical methods

Participants were categorized based on tertile cut‐off points of DAQS. One‐way ANOVA for continuous variables and chi‐square analysis for categorical variables were used to compare general characteristics and dietary intake across tertile of DAQS. Age‐ and energy‐adjusted intakes and food and nutrient consumption across tertile of DAQS were calculated using ANCOVA. To examine the association between DAQS and MetS and its components, we used multivariable logistic regression analysis in different models. First, we controlled for the confounding effect of age and sex. In the second model, we further adjusted for cigarette smoking, physical activity, and socio‐economic status and BMI. The overall trend of odds ratios across quartiles of DAQS was calculated by considering the median of DAQS in each quartile as a continuous variable. All statistical analyses were performed using the Statistical Package for the Social Sciences (SPSS version 25; SPSS Inc.); we considered *p* < .05 as the significance level.

## 3 RESULTS

The general characteristics of the participants by tertiles of DAQSs are shown in Table 1. A total of 270 participants (43.7% men; 56.3% women) were included in this study. The mean age of participants was 36.77 ± 13.19 years with a mean BMI of 25.62 kg/m2. Compared with females in the lowest tertile, those in the highest tertile of the DAQS had significantly higher FM, weight, BMI, and WC (P‐value = 0.01 for all comparisons).

**Table 1:** General characteristics of study participants by tertiles of DAQS

	All Mean ± *SD* or%	tertiles of DAQS							
		Males (43.7%)			P_value_	Females (56.3%)			P_value_
		T1 (≤1)	T2 (1–2)	T3 (≥3)		T1 (≤1)	T2 (1–2)	T3 (≥3)	
*N*	270	10	41	67		23	91	38	
Height (cm)	168 ± 9.96	176 ± 2.63	177 ± 7.91	175 ± 7.51	0.63	160 ± 6.90	161 ± 6.59	162 ± 5.42	0.74
Age(year)	36.7 ± 13.1	35.5 ± 12.5	36.0 ± 12.7	39.7 ± 13.3	0.30	33.4 ± 13.9	35.5 ± 12.5	37.6 ± 14.5	0.47
FFM(kg)	50.1 ± 12.8	59.3 ± 5.19	61.8 ± 10.0	61.6 ± 9.04	0.73	40.6 ± 6.35	41.1 ± 5.41	42.1 ± 8.37	0.64
FM(kg)	22.4 ± 9.38	18.4 ± 7.78	19.7 ± 9.08	21.5 ± 9.52	0.44	23.5 ± 8.76	22.3 ± 7.85	27.7 ± 11.7	0.01
Weight (kg)	72.7 ± 16.0	77.7 ± 11.4	81.6 ± 15.9	83.0 ± 14.1	0.55	64.1 ± 13.0	63.5 ± 11.2	70.9 ± 16.1	0.01
WC (cm)	89.6 ± 12.5	90.9 ± 10.4	92.5 ± 12.5	95.2 ± 12.5	0.39	85.1 ± 11.8	84.6 ± 10.3	91.1 ± 13.6	0.01
WHR	0.90 ± 0.06	0.91 ± 0.05	0.91 ± 0.06	0.92 ± 0.07	0.44	0.88 ± 0.05	0.89 ± 0.05	0.90 ± 0.06	0.05
BMI (kg/m^2^)	25.6 ± 4.66	25.0 ± 3.92	25.8 ± 4.06	26.8 ± 4.11	0.27	24.8 ± 4.95	24.2 ± 4.20	27.0 ± 6.27	0.01
Marital status(%)					0.24				0.18
Single	46.8	5.0	17.8	22.8		10.0	27.8	10.0	
**Married**	53.2	3.4	16.9	33.9		4.6	32.5	15.2	
Smoking(%)					0.25				0.23
**Nonsmoker**	86.6	8.5	29.7	35.6		13.9	59.6	23.2	
**Former and current smoker**	13.4	0.0	5.0	21.2		0.7	0.7	2.0	
Physical activity(%)					0.23				0.21
**Low**	38.3	3.4	11.9	16.1		4.6	30.5	8.6	
**Medium**	41.3	5.1	12.7	20.3		8.6	23.2	11.9	
**High**	20.4	0.0	10.2	20.3		1.3	6.6	4.6	
Diabetes(%)					0.70				0.12
**Yes**	3.3	0.0	0.8	2.5		1.3	0.7	1.3	
**No**	96.7	8.5	33.9	54.2		13.2	59.6	23.8	
CVD(%)					0.71				0.32
**Yes**	2.2	0.0	0.8	2.5		0.7	0.7	0.0	
**No**	97.8	8.5	33.9	54.2		14.0	60.0	24.7	

Note

P‐value less than 0.05 was considered significant. P‐value less than 0.05 was considered significant. Values are based on average ± standard deviation or reported percentage. One‐way ANOVA for quantitative data and Chi‐2 test for qualitative data have been used. Subjects in the first tertile of DAQS had DAQs score between (≤1); second tertile: between (1–2); third tertile: between (≥3).

Abbreviations: BMI, body mass index; FFM, fat‐free mass; FM, fat mass; WC, waist circumference; WHR, waist‐hip ratio.

Table 2 presents dietary intake of nutrients according to the tertiles of the DAQS. Adherence to DAQS showed a significant increase for intake of vitamin B6 (P‐value = 0.02), riboflavin (P‐value < 0.001), folate (P‐value = 0.03), selenium (P‐value = 0.03), vitamin D (P‐value < 0.001), and calcium (P‐value < 0.001). Compared with participants in the lowest tertile, those in the highest tertile of the DAQS had significantly lower intakes of carbohydrates (P‐value < 0.01), thiamin (P‐value < 0.001) and higher intakes of magnesium (P‐value < 0.01), vitamin C (P‐value = 0.02), zinc, protein, vitamin A, and energy (P‐value < 0.001 for all).

**Table 2:** Dietary intake of nutrients according to the tertiles (T) of the DAQS

	All = 270 Mean ± *SD*	Tertiles of DAQS			P _value_	P _trend_
		T1 (*n* = 34)	T2 (*n* = 132)	T3 (*n* = 104)		
Energy (1000kcal/d)	2.39 ± 0.96	1.44 ± 0.36	2.03 ± 0.55	3.11 ± 10.0	<0.001	<0.001
Carbohydrates (g/d/1000kcal)	142 ± 20.1	149 ± 60.88	144 ± 19.3	137 ± 20.5	<0.01	<0.01
Protein (g/d/1000kcal)	38.2 ± 8.82	35.7 ± 7.10	35.6 ± 6.82	42.0 ± 10.0	<0.001	<0.001
Total fat (g/d/1000kcal)	33.2 ± 8.35	31.3 ± 7.56	33.4 ± 7.62	33.4 ± 7.62	0.41	0.22
Thiamin (mg) (mg/d/1000kcal)	0.78 ± 0.16	0.84 ± 0.12	0.79 ± 0.16	0.75 ± 0.15	<0.001	<0.001
Riboflavin (mg/d/1000kcal)	0.73 ± 0.21	0.64 ± 0.16	0.68 ± 0.17	0.82 ± 0.24	<0.001	<0.001
Niacin (mg/d/1000kcal)	9.37 ± 1.82	9.49 ± 1.65	9.11 ± 1.70	9.67 ± 1.98	0.06	0.64
Vitamin B6 (mg/d/1000kcal)	0.62 ± 0.19	0.56 ± 0.16	0.61 ± 0.19	0.66 ± 0.20	0.02	0.01
Folate (µg/d/1000kcal)	133 ± 38.8	126 ± 32.2	129 ± 39.2	141 ± 39.3	0.03	0.07
Vitamin D (µg/d/1000kcal)	0.97 ± 0.83	0.73 ± 0.51	0.82 ± 0.60	1.24 ± 1.07	<0.001	<0.01
Vitamin E (mg/d/1000kcal)	1.89 ± 1.03	1.73 ± 0.46	1.78 ± 0.60	2.07 ± 1.47	0.07	0.10
Vitamin A (µg/d/1000kcal)	582 ± 401	316 ± 63.3	632 ± 480	598 ± 311	<0.001	<0.001
Vitamin C(mg/d/1000kcal)	60.0 ± 28.3	49.6 ± 18.9	64.0 ± 30.6	58.1 ± 26.7	0.02	0.13
Zn (mg/d/1000kcal)	4.09 ± 1.03	3.91 ± 0.85	3.71 ± 0.87	4.63 ± 1.05	<0.001	<0.001
Se (µg/d/1000kcal)	0.02 ± 0.01	0.02 ± 0.01	0.01 ± 0.01	0.02 ± 0.01	0.03	0.07
Fe (mg/d/1000kcal)	9.21 ± 3.16	10.1 ± 3.73	9.10 ± 2.91	9.07 ± 3.28	0.21	0.09
Ca (mg/d/1000kcal)	433 ± 152	376 ± 76.8	409 ± 125	481 ± 184	<0.001	<0.01
Magnesium (mg/d/1000kcal)	122 ± 24.0	119 ± 19.0	117 ± 25.1	128 ± 22.9	<0.01	0.08

Note

P‐value less than 0.05 was considered significant. Values are based on mean ± standard deviation. P_value_ obtained from one‐way ANOVA test.

Abbreviation: DAQS: dietary antioxidant quality score.

Daily intake of the nutrients in the study population is shown in Table 3. Men had lower daily intake of folate (P‐value = 0.04), vitamin D, vitamin E, and magnesium (P‐value < 0.001 for all) than Dietary Reference Intake. Women also had lower intake of folate, vitamin D, vitamin E, calcium, selenium, and magnesium (P‐value < 0.001 for all) than Dietary Reference Intake. The multivariate adjusted means for TG, SBP, DBP, FBS, HDL, and WC according to tertiles of DAQS indicated in Table 4. Adherence to DAQS showed a nonsignificant decrease for DBP (P‐value = 0.4), SBP (P‐value = 0.2), and HDL (P‐value = 0.4). A nonsignificant increase was found for TG (P‐value = 0.9) across tertiles of DAQS. Results were similar after adjustment for confounding factors, including age, sex, physical activity, smoking, marital status, energy, and BMI. Multivariate‐adjusted odds ratios and 95% confidence intervals for metabolic syndrome across tertiles of DAQS are shown in Table 5. Adherence to DAQS showed a significant decrease for odds of SBP (OR: 0.17, 95%CI: (0.04, 0.65), P‐value = 0.03). After adjusting for potential confounders, the association remained unchanged (P_ANCOVA_ = 0.03). The risk of TG, SBP, DBP, FBS, and WC was not changed significantly after control for confounders across tertiles of DAQS.

**Table 3:** Daily intake of the nutrients in the study population

	**Males**	**Females**
	**% sample under DRI**	**% sample under DRI**
Thiamin (mg/d)	8.5	18.5
Riboflavin (mg/d)	27.4	29.8
Niacin (mg/d)	16.2	25.2
Vitamin B6 (mg/d)	39.3	55.6
Folate (µg/d)	77.8	86.1
Vitamin D (µg/d)	37.6	49.7
Vitamin E (mg/d)	38.3	43.1
Vitamin A (µg/d)	34.2	26.5
Vitamin C (mg/d)	24.8	22.5
Zn (mg/d)	58.1	53.6
Se (µg/d)	53.8	74.2
Fe (mg/d)	20.1	53.0
Ca (mg/d)	45.3	63.6
Magnesium (mg/d)	83.2	76.8

Note

Values are based on percentage

Abbreviations: µg, microgram; d, day; DRI, Dietary Reference Intake; mg, milligram.

**Table 4:** The Multivariate adjusted means for TG, SBP, DBP, FBS, HDL, and WC *according to tertiles of DAQS*

	All = 271 Mean ± *SD*	Tertiles of DAQS			P1	P2	P3
		T1 = 34	T2 = 132	T3 = 105			
TG(mg/dl)	119 ± 69.4	115 ± 53.7	119 ± 74.2	121 ± 68	0.92	0.69	0.86
SBP(mmHg)	111 ± 19.1	112 ± 24.7	113 ± 16	109 ± 20.7	0.27	0.16	0.65
DBP(mmHg)	70.6 ± 10.6	72.2 ± 11.6	70.8 ± 10.3	69.8 ± 10.6	0.48	0.23	0.62
FBS(mg/dl)	98.3 ± 18.7	99.2 ± 11.4	97.7 ± 12.9	98.9 ± 25.7	0.84	0.87	0.72
HDL(mg/dl)	49.7 ± 10.6	50.4 ± 10.1	50.4 ± 11	48.7 ± 10.3	0.42	0.25	0.33
WC(cm)	89.6 ± 12.5	89.4 ± 11.4	90.2 ± 13.3	88.8 ± 11.9	0.69	0.59	0.94

Note

P1 ANOVA test. P2 p trend. P3 ANCOVA test adjusted for age, sex, physical activity, smoking, marital status, energy, and BMI.

Abbreviations: cm, centimeter; DBP, diastolic blood pressure; dl, deciliter; FBS, fasting blood sugar; HDL, high‐density cholesterol; mg, milligram; mmHg, millimeter of mercury; SBP, systolic blood pressure; TG, triglyceride; WC, waist circumference.

**Table 5:** multivariate‐adjusted odds ratios and 95% confidence intervals for metabolic syndrome across tertiles (T) of DAQS

	Tertiles of DAQS	P _value_		
	T1 = 34	T2 = 132	T3 = 105	
MetS				
Model 1	1.00	1.92(0.62,5.94)	1.76(0.55,5.58)	0.52
Model 2	1.00	1.54(0.47,5.04)	1.73(0.51,5.84)	0.67
Elevated TG (mg/dL)				
Model 1	1.00	1.85(0.66,5.19)	1.90(0.67,5.44)	0.45
Model 2	1.00	1.72(0.60,4.94)	1.95(0.67,5.71)	0.46
Elevated SBP ( mmHg)				
Model 1	1.00	0.54(0.21,1.38)	0.23(0.07,0.69)	0.03
Model 2	1.00	0.32(0.10,0.99)	0.17(0.04,0.65)	0.03
Elevated DBP ( mmHg)				
Model 1	1.00	0.57(0.14,2.36)	0.62(0.14,2.65)	0.74
Model 2	1.00	0.39(0.08,1.81)	0.55(0.11,2.62)	0.48
Elevated FBS (mg/dL)				
Model 1	1.00	0.92(0.42,2.00)	0.77(0.34,1.72)	0.74
Model 2	1.00	0.71(0.31,1.61)	0.67(0.29,1.56)	0.65
Low HDL (mg/dL)				
Model 1	1.00	1.17(0.53,2.58)	1.21(0.54,2.72)	0.89
Model 2	1.00	0.91(0.38,2.13)	1.09(0.45,2.61)	0.82
High WC (cm)				
Model 1	1.00	1.07(0.47,2.39)	0.91(0.39,2.10)	0.85
Model 2	1.00	1.03(0.20,5.34)	1.53(0.29,8.12)	0.68

Note

Model 1: Crude. Model 2: adjusted by age, sex, physical activity, smoking, marital status, energy, and BMI.

Abbreviations: DBP, diastolic blood pressure; FBS, fasting blood sugar; HDL‐C, high‐density lipoprotein cholesterol; SBP, systolic blood pressure; TG, triglyceride; WC, waist circumference.

## 4 DISCUSSION

This study aimed to examine the association of DAQS with MetS among Tehran's adult population. Adherence to DAQS showed a significant decrease for SBP. We also found that the overall DAQS were not significantly related to MetS and its other components. Central obesity, insulin resistance, dyslipidemia, and hypertension are the main components of metabolic syndrome (MetS) that is coincident with unhealthy dietary patterns in the Middle‐Eastern countries (Rezagholizadeh et al., 2017; Shab‐Bidar et al., 2014). Lorzadeh et al indicated that dietary habits associated with metabolic syndrome in a sample of Iranian adults (Lorzadeh et al., 2020). Their study revealed that eating breakfast has an inverse relationship with metabolic syndrome (Lorzadeh et al., 2020).

Our results are in line with the results of other studies such as Chen et al that indicated antioxidant vitamins may be the underlying cause to prevent hypertension (Chen et al., 2002). Waśkiewicz et al observed high antioxidant food consumption in Polish adults was associated with lower risks of hypertension (Waśkiewicz et al., 2019). Moreover, Hassani Zadeh et al indicated a significant relationship between moderate adherence to healthy eating index (HEI) and fasting blood glucose in both men and women. They also showed that moderate adherence to healthy dietary pattern decreased the prevalence of MetS in women(Zadeh et al., 2020; Zadeh et al., 2020). Rodrigo et al also found a strong association between blood pressure and some oxidative stress parameters and showed a possible role of oxidative stress in essential hypertension pathophysiology (Rodrigo et al., 2007). ROS exposure enhances the activity of antioxidant enzymes. Therefore, genes encoding these enzymes are coordinately controlled in their regulatory regions by the antioxidant responsive elements (ARE), a mechanism that occurs through the activation of the transcription factor NF‐E2–associated factor 2 (Nrf2). Binding Nrf2 to these ARE sites results in up‐regulation of downstream genes which regulate the activity of antioxidant enzymes in order to compensate for the toxicity of ROS. In most hypertensives, this mechanism may be enabled for response to their ROS levels (Bae et al., 2004; Lee & Johnson, 2004; Talalay et al., 2003). In addition, we found that the total DAQS were not related significantly to MetS. In longitudinal supplementations with nutritional doses of antioxidant vitamins and minerals, no beneficial effects on fasting blood glucose and the risk of hypertension and MetS and adverse effects on lipid profiles were observed (Czernichow et al., 2006, 2009; Shahavandi, Djafari, et al., 2020). The results of some studies, though, are inconsistent with our study results. Puchau et al suggested that higher intakes of total antioxidant could be a potential risk factor of developing MetS characteristics (Puchau et al., 2010). Sharma et al indicated a strong positive association was observed in MetS between oxidative stress and insulin resistance(P. Sharma et al., 2005). All the individual components and the onset of cardiovascular complications in subjects with MetS were associated with oxidative stress(Furukawa et al., 2017; Keaney Jr et al., 2003). Although it is generally accepted that the primary pathogenic mechanism causing the first stage of metabolic changes in patients with the MetS is focused on insulin resistance, there was an abundance of evidence suggesting a close relationship between the MetS, a chronic low‐level inflammation and oxidative stress as second‐level abnormalities (Wellen & Hotamisligil, 2005). Oxidative stress potentially plays an important role in the pathogenesis of vascular modifications by either triggering or exacerbating the biochemical processes that follow the metabolic syndrome (Stocker & Keaney Jr, 2004). Although some of the component characteristics of the MetS are understood to share common pathogenic mechanisms of damage the effect of genetic predisposition and gene expression control, and the function of the environment and dietary pattern in determining inflammatory process‐triggered oxidation, remains unclear (Sahaf, Heydari, Herzenberg, & Herzenberg, 2005; Vendemiale et al., 1995). Such aspects of the issue deserve special attention since it is suspected that oxidative stress may be amplified in patients with the MetS by a concomitant antioxidant deficit that may support the propagation of oxidative modifications from intracellular to extracellular spaces and from confined to distant sites resulting in a systemic oxidative stress state (Sahaf et al., 2005; Vendemiale et al., 1995). One explanation is that there was insufficient variation in antioxidant intakes across tertiles of DAQS. Second, because of the temporal relationship between the measured exposure and the outcome, a relationship could remain undetected that did not cover the true latent period. Third, unmeasured variables exist which we did not control to influence the antioxidant–MetS relationship. Fourth, the differences observed in our study, as opposed to other studies, may be due to the cross‐sectional design which prevents causal inferences to be made. Moreover, the small number of participants in our study may be another reason for nonsignificant results, although we had enough power to detect the diet–disease relationship. We believe that there were many strengths in our study. The study looked at a wide range of antioxidants. For the collection of dietary data, a validated FFQ was implemented. Some limitations of the present study should be considered; participants’' usual dietary intakes were only assessed at baseline, whereas several dietary intake evaluations could have increased the validity of the results. The small sample size was the main limitation of statistical analytical ability. However, although we have adjusted for possible confounding factors, there may be residual or unmediated confounding. No assessment was made of the dietary patterns of other antioxidants, such as flavonoids.

## 5 CONCLUSION

Although we observed an improvement in SBP with greater adherence to dietary antioxidant quality score, there was no association between DAQS and metabolic syndrome and its other components. This study provides more reasons to suggest foods rich in antioxidants as a useful tool for promoting health and avoiding disease. Besides the current guidelines recommending increased consumption of whole‐plant foods, it would definitely be more important to concentrate on food selection focused on the antioxidant content of products.

## 6 CONFLICT OF INTEREST

The authors declared no conflicts of interest.

## 7 AUTHOR CONTRIBUTIONS

MSH and SS‐b contributed in conception, design, search, data interpretation, and manuscript drafting. HSH, NP, and FS contributed in design, data interpretation, statistical analyses, and manuscript drafting. KDj contributed in conception, design, and statistical analyses. SS‐b supervised the study. All authors read and approved the final manuscript.

8

## Data Availability

Raw data were generated at Tehran University of Medical Sciences. Derived data supporting the findings of this study are available from the corresponding author SS‐b on request.
